# Preparation, Characterization, and Biological Features of Cactus Coated Bacterial Cellulose Hydrogels

**DOI:** 10.3390/gels8020088

**Published:** 2022-01-30

**Authors:** Tahseen Kamal, Mazhar Ul-Islam, Sher Bahadar Khan, Esraa M. Bakhsh, Muhammad Tariq Saeed Chani

**Affiliations:** 1 Center of Excellence for Advanced Materials Research, King Abdulaziz University, Jeddah 21589, Saudi Arabia; sbkhan@kau.edu.sa (S.B.K.); mtmohamad@kau.edu.sa (M.T.S.C.); 2 Department of Chemical Engineering, College of Engineering, Dhofar University, Salalah 211, Oman; mulislam@du.edu.om; 3 Department of Chemistry, King Abdulaziz University, Jeddah 80200, Saudi Arabia; ibakhsh@kau.edu.sa

**Keywords:** bacterial cellulose, adsorption, cactus composite, mechanical properties, biocompatibility

## Abstract

The current study was aimed at developing BC-Cactus (BCC) composite hydrogels with impressive mechanical features for their potential applications in medical and environmental sectors. BCC composites hydrogels were developed through cactus gel coating on a never dried BC matrix. The FE-SEM micrographs confirmed the saturation of BC fibrils with cactus gel. Additionally, the presence of various functional groups and alteration in crystalline behavior was confirmed through FTIR and XRD analysis. Mechanical testing illustrated a three-times increase in the strain failure and an increase of 1.6 times in the tensile strength of BCC composite. Absorption capabilities of BCC were much higher than pure BC and it retained water for a longer period of time. Additionally, the rewetting and absorption potentials of composites were also higher than pure BC. The composite efficiently adsorbed Pb, Zn, Cu, and Co metals. Biocompatibility studies against human HaCat cell line indicated much better cell adhesion and proliferation of BCC compared to BC. These findings advocate that the BCC composite could find applications in medical, pharmaceutical and environmental fields.

## 1. Introduction

Bacterial cellulose (BC) is an organic polymer synthesized by a class of acetic acid producing bacterial strains [1]. Cell-free systems can also produce BC by using different sugar sources [2,3]. These cell free systems produce glucose chains, which self-aggregate and form protruding cellulose nanofibers [4]. These cellulose nanofibers give rise to a web-shaped three-dimensional nano-porous structure with a high surface area. The nano-porous geometry, hydrophilicity, and presence of hydroxyl groups make BC an excellent matrix for holding liquids [5]. In addition to this, the structural, mechanical, physico-chemical, and biological properties of BC has made it an important entity in biomedical [6,7], environmental [5], pharmaceutical [8,9], sensing [10], energy [11], and several other fields. Although BC has found its applications in varied sectors, due to its low mechanical strength and lack of adhesion sites, its application is restricted in many fields. Based on the background of BC, the need to develop its composites with other materials to improve its existing features is of the upmost importance. Incorporation of BC with therapeutically active and biocompatible natural compounds/extracts has proved to be an effective approach towards the synthesis of BC composites with enhanced therapeutic potential and mechanical strength.

The cactus *Opuntia* (genus *Opuntia*, family *Cactaceae*) is a xerophytic plant. This plant is mainly grown in an arid and semi-arid region. Morphologically, Cactus plants are composed of flattened stems (cladodes), fruits and the areoles having minute-barbed spines, rudimentary leaves on new pads and seeds [12]. The vegetative part, frequently called as pads or cladodes are succulent and serves the purpose of water storage. Cactus plants are known to retain water due to high mucilage production in cladodes and fruits [13]. The epidermis of cladodes has two layers, the chlorenchyma (green cells), and an internal layer comprised of parenchyma (cylinder of white cells), which are mainly responsible for water storage. The chlorenchyma and parenchyma contain mucilaginous cells that store mucilage, which exhibits the osmotic property of strong water retention.

*Opuntia* spp. has been used traditionally in food and as well for medicinal purposes. Their extracts have been found to possess anti-inflammatory and analgesic properties [14] and exhibit hypoglycemic effects [15]. In addition to its medicinal properties, mucilage obtained from pads is a potential source of hydrocolloids, which are useful in cosmetic applications [12]. Some studies have reported its use in water purification or filtration [16]. *Opuntia* spp. extract is also reported to increase the plasticity and water absorption capacity of mortar [17].

In the present study, we have synthesized a composite of BC with Cactus pad extract through a simple coating technique. The aim was to enhance the BC existing mechanical, liquid holding and biocompatible features. The synthesis of BC-Cactus hydrogel was investigated through various analytical tools and its mechanical, adsorption and biocompatible activities were investigated against pure BC as control.

## 2. Results and Discussion

### 2.1. Composite Synthesis and Characterization

**Figure 1:**
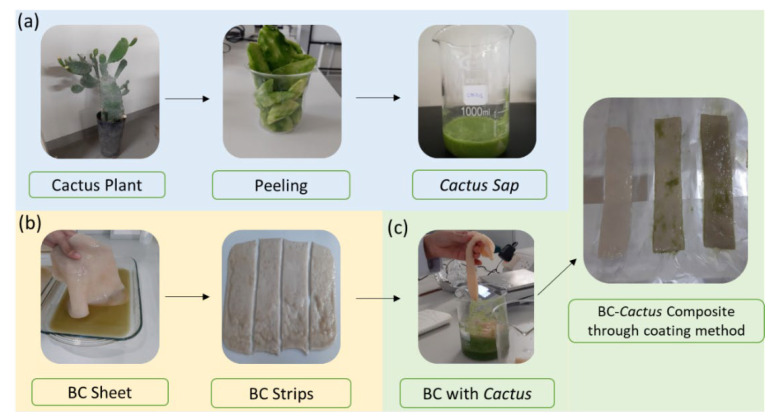
Different steps for the preparation of (**a**) cactus sap, (**b**) BC strips and (**c**) BCC composite.

Among the number of strategies used to develop BC composites, the surface coating is preferred due to the fact that it retains the basic morphological features of BC and augment it with additional ones coming from the functional coating. Materials addition in the BC production media for developing BC composites through in situ strategy can enhance the viscosity that, in turn, can retard the microbial activities and BC sheet formations [18]. BC composites with plant extracts, and gels including aloe vera hydrogel have been developed through ex-situ approaches [19]. The immersion of never dried BC sheets in liquid or semi liquid media offer ideal environment for solution and gels to attach to the fibril surface along with impregnating in the porous BC matrix. The BCC composite synthesis process through surface coating strategy has been illustrated in Figure 1.

**Figure 2:**
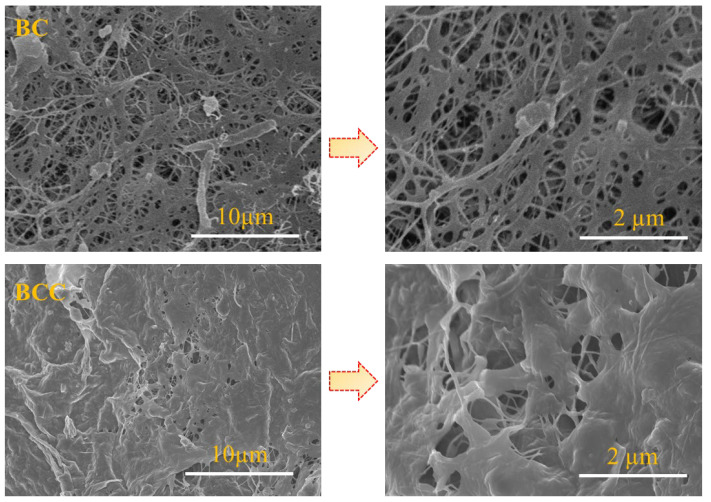
Field-emission scanning electron micrographs of surface morphologies of pristine BC and BCC composites.

The morphology of BC after treatment with Cactus gel was observed through FE-SEM. The micrographs shown in Figure 2 indicate the porous fibril structure of BC. The thickness of fibril was below 100 nm and these were well arranged in 3D network form. While looking at the micrographs of BCC composite, it was clear that BC surface was covered by the Cactus gel. The porosity of native BC is much reduced. Hydroxyl moieties of BC offer an ideal situation for the attachment of various compounds to BC fibers through hydrogen bonding interactions. Strong attachment and covering of BC surface by Cactus is expected to affect its phytochemical and mechanical properties.

**Figure 3:**
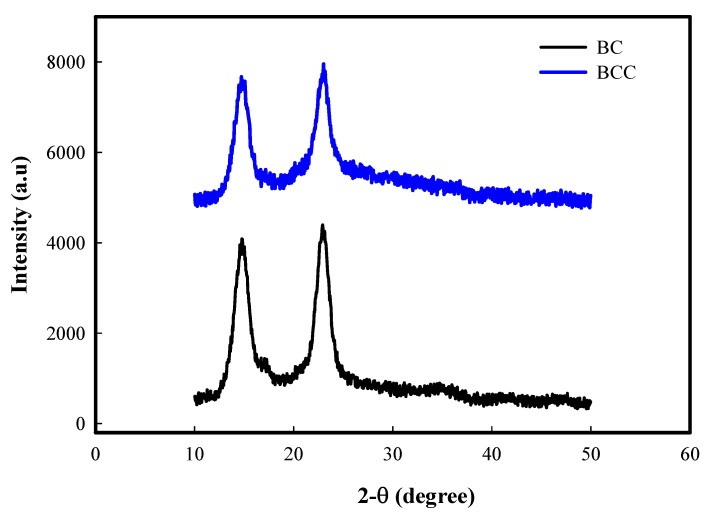
XRD analysis of BC, and BCC composite films.

XRD is a significant tool to study the crystalline nature of pure compounds and structural modifications observed via developing their composites. XRD results of the BC and BCC composites are shown in Figure 3. Native BC is semi-crystalline polymer consisting of both crystalline and amorphous parts. BC normally represents two main crystallinic peaks appearing at 2θ 14.6° and 22.7°) corresponding to the crystallo-graphic planes of (110) and (200), respectively, as apparent in the current study (Figure 3). Another small peak occasionally appears at 16.2°. An amorphous halo present at 2θ 19.6° represents the amorphous part of the pure BC [20]. Depending upon the nature of additive, the BC composite illustrates additional crystallinic peaks as well as modification in BC own crystallinic peak intensities. The XRD spectra of BCC shown in Figure 3 did not indicate any additional peak that is based on the non-crystalline nature of Cactus. However, one can find the intensities of crystalline BC have reduced to some extent as compared to pure BC. This intern reflects that BCC have less crystallinity than BC, which support the finding of mechanical testing where a huge increase in the stretching behavior of BCC was observed.

### 2.2. Mechanical Strength of BC and BCC Composite Hydrogel

**Figure 4:**
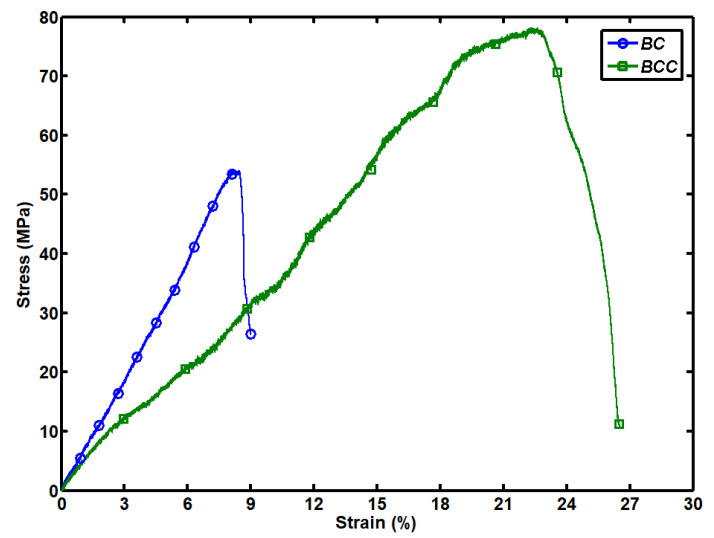
Stress–strain curve of pristine BC and BCC composite.

**Figure 5:**
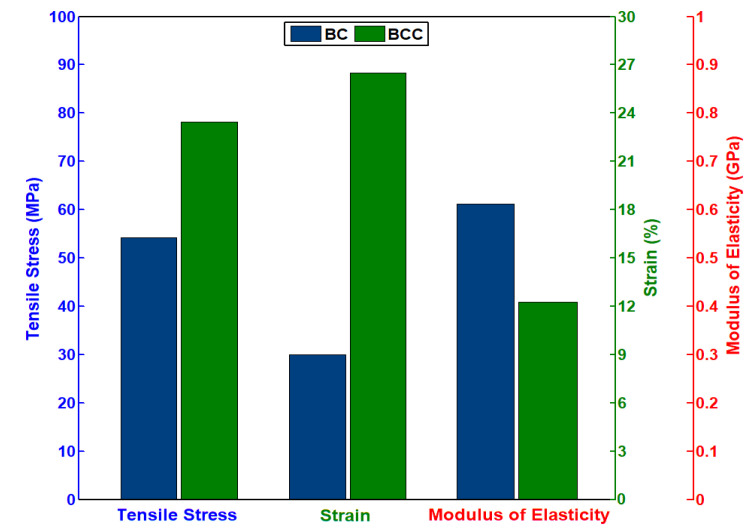
Tensile strength, strain (%), and modulus of elasticity of BC and BCC composite.

The tensile testing results for the pure BC and BCC are shown in Figure 4. Stress–strain curves for both samples move linearly toward their peak point and as the fracture started in the samples, the curves started to drop down. Stress–strain curves indicate that both samples are brittle in nature. Pure BC sample shows the maximum tensile strength of 54.14 MPa and failure strain of 9 %, by adding the Cactus coating on the pure BC its tensile strength and elongation properties increase as shown for the BCC, which have a maximum strength and failure strain of 78.18 MPa and 26.52%, respectively, as shown in Figure 5. Tensile strength of the BCC sample increase by 1.5 times and failure strain three times compared to the pure BC sample. Young’s modulus of the BCC sample is 0.4 MPa, which is less than the Young’s modulus of BC that is 0.6 MPa. Overall mechanical properties of pure BC are increased by impregnating cactus gel in its structure. The BC composites have been reported to show much higher mechanical features including tensile strength, modulus of elasticity or both depending upon the additives nature [21,22]. Cactus mechanical properties, specifically elastic features, are well explored. Some studies have reported very high modulus of elasticity (around 30 GPa) obtained with dry and wet cactus spines [23]. Developing BCC through such approach not only protects the existing strength of BC, but also improves its strength [18]. Earlier development of BC composites with graphene oxide has depicted an increase in mechanical strength [24]. In situ composites on the other hand interfere with BC fibril structure during the synthetic process, which can cause a reduction in its mechanical, crystalline and physiological features. It is expected that BCC composites with high elastic feature can find impressive applications in medical, cosmetics textile and other fields.

### 2.3. Absorption Studies

**Figure 6:**
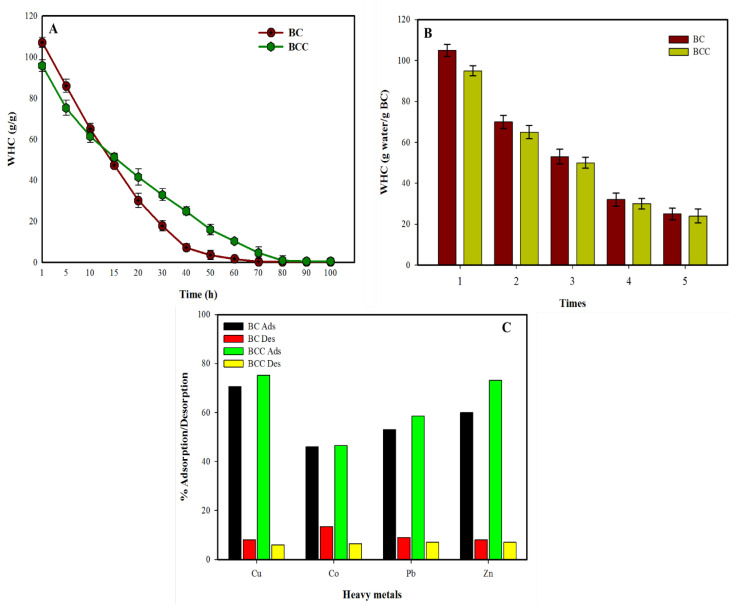
Absorption analysis of BC and BCC, (**A**) Water holding capacity (**B**) WHC in repeated batches through drying and re-swelling processes, (**C**) heavy metal absorption analysis.

High porosity and hydrophilic nature bless BC with remarkable absorbing capabilities. BC and its composite applications in medical fields are mainly built on its high absorbing capabilities besides its nontoxic and biocompatible nature [1]. It has been well established that never dried BC may absorb water over 100 times of its weight. Pore size and volume of BC indeed play vital role in defining the liquid holding capacities of BC samples. It has been reported that never dried BC has very high porosity and similarly high WHC. During drying process, the removal of surface water leads to the pore closure and conversion of reversible hydrogen bindings to irreversible hydrogen bindings. Therefore, we observed that water holding capabilities of dried and re-swelled BC are always less than never dried BC sheets. Another important aspect we observed is that freeze-dried BC results in better porosity than air-dried BC [25]. Water holding and retention capabilities of BC and BCC indicated in Figure 6A illustrated that pure BC hold around 109 times water of its dry weight compared to BCC, which could hold around 94 times. These results supported the SEM observation that cactus gel impregnated in BC network had reduced the porosity to some extent. While considering the water retention time of both BC and BCC, it was observed that BCC could retain water for a longer time compared to pure BC. Figure 6B illustrates that water lost from BC was much quicker and the curve was much more linear until almost a complete loss of water. BCC on other hand indicated reduced trends of water loss and retained a reasonable quantity of water until the extended time frame of 70 h. The slow release could be attributed to the interaction of cactus gel with bonded water and relatively lower porosity of BC composites that hindered the escape of water molecules. The extension of WHC analysis in repeated batches of the same samples indicated that every time the WHC values were reduced to a great extent, around 30% reduction in WHC was observed in between the never dried and 1st dried and re-swelled samples. The WHC kept on reducing with every drying and re-swelling cycle, however, it was important to observe that even after the fifth re-swelling cycle process, BC and BCC were able to hold more than 20% water of their dry weight. High WHC and slow WRR from BCC hydrogels are significant features for their applicability in medical and environmental fields [26,27].

The Nanofibrillar structure and porous geometry of BC significantly contribute to the absorption of heavy metals [28]. BC and BC composite-based filters have been developed for absorbing unwanted toxic materials from industrial effluents and wastewater. The results of heavy metal absorbing proficiencies of BC and BCC observed against Cobalt (Co), Copper (Cu), Lead (Pb) and Zinc (Zn) are shown in Figure 6C. The results indicated that both BC and BCC were able to absorb substantial amount of heavy metals from water. Overall BCC indicated better absorption capabilities compared to BC. The development of BC composites for heavy metal adsorption have been reported earlier. Several synthetic polymers including chitosan, polyethylene glycol, attapulgite and other polymers for removal of Cu, Pb, Cr and other heavy metals. The use of natural materials like cactus gel for such applications have been rarely explored. Considering the exceptional mechanical, absorbing, and heavy metal removal feasibilities, it can be stated that BC composites with natural polymers and other compounds could be of vital importance in medical and environmental applications.

### 2.4. Biocompatibility of BCC Composite Hydrogel

**Figure 7:**
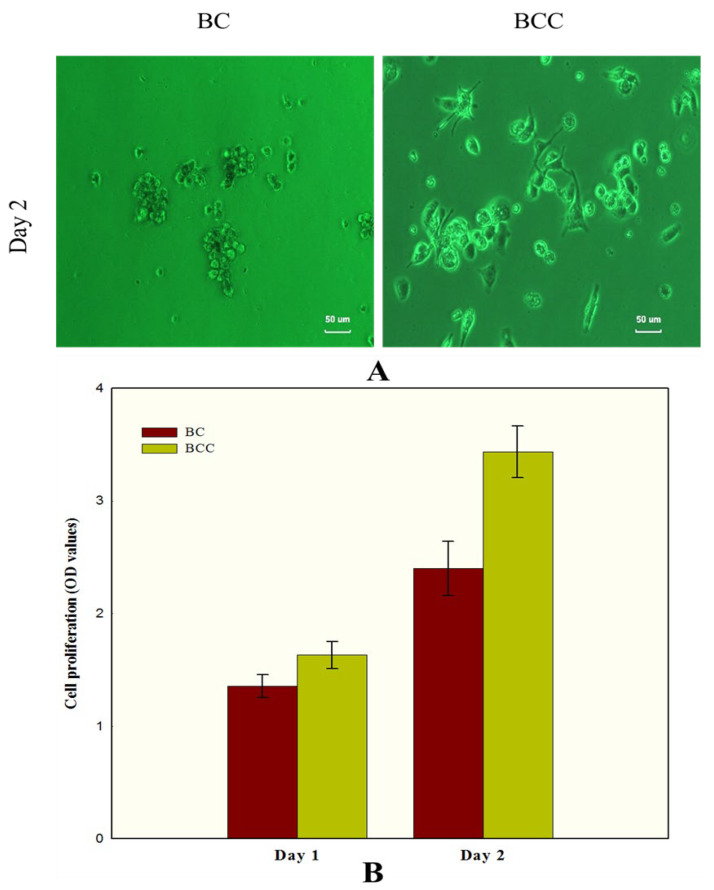
(**A**) Morphology observation of human keratinocytes (HaCaT) adhesion on BC and BCC composites on day 2. (**B**) Cellular proliferations on both BC and BCC at day 1 and day 2.

Biocompatible materials are widely employed to evaluate the various cellular processes. The attachment and spreading of animal cells on the materials surface provide a perception of its biocompatibility. BC has been widely explored for its potential biocompatible features and consequent medical applications. It has been observed that pure BC exhibit low to moderate levels of biocompatibility, which have indeed been improved by the development of its composites with various polymers and natural materials [29,30]. Herein, the biocompatible features of BC and BCC were observed against HaCaT cell lines. The morphological results obtained through surface phase-contrast microscopic analysis illustrated that cell lines were strongly attached to the BCC surface as compared to pure BC (Figure 7). Furthermore, the HaCaT cell lines distribution on BC surface was much variable as compared to BCC, where the surface was relatively smooth and well covered. This better attachment of the cells on the BCC surface can be linked to the additive effect of the nontoxic, and biocompatible properties of cactus. On the contrary, the cells attached on the native BC surface displayed strong cell-cell interaction compared to BCC cell-scaffold interaction that led to cell aggregation. (Figure 7A). The cell proliferation results revealed that the cell on BCC demonstrated high viability after 2 days’ culture (Figure 7B), in comparison to pure BC. The BCC offers better spreading on the surface and high cell to scaffold interaction, whereas BC indicates higher cell to cell interaction and less spreading of the cell. These results provide an insight that, besides enhancing the mechanical features, the high biocompatible features of BCC can offer better applications in medical fields.

## 3. Conclusions

BC composites with cactus gel (BCC) were successfully developed by impregnating cactus gel in a never dried BC matrix. Structural analysis indicated the formation of a cactus layer on the BC surface, whereas the bonding and crystalline features were further validated through FTIR and XRD studies. BCC composite illustrated much higher mechanical strength and three times higher modulus of elasticity. The biocompatibility evaluation against human HaCat cells showed much better cell attachment and proliferation abilities on the BCC surface. Additionally, the composite exhibited longer liquid retention capabilities along with better heavy metal absorption proportions. It can be concluded that the developed BCC composites with advanced biocompatible, mechanical, and absorption features could be used for applications in biomedical, pharmaceutical, and environment fields. 

## 4. Experimentation

### 4.1. Microbial Cell Culture and BC Production

*Gluconacetobacter hansenii* was used as a BC-producing strain. This research followed an established protocol [31] for the cell culture and BC production.

### 4.2. Production of BCC

*Opuntia* spp. (Cactus) plants were collected from Salalah, Oman. The mature fresh pads or cladodes were cut from the plants and washed with water to remove dust. The thick epidermis of cladodes was gently peeled and inner parenchymous cells were crushed to collect the semi-solid mucilaginous content and kept in a sterile beaker. After that, BC and Cactus (BCC) hydrogel composite were prepared by dipping the BC sheets in Cactus gel for 2–3 days, as a result of which the gel was adsorbed on the surface and internal matrix of BC samples. The Cactus semi-solid gel-loaded BC (BC-Cactus *or BCC*) samples were separated from the beaker, gently cleaned, and stored for further use.

### 4.3. Characterization

The morphology of BC and BCC composite was observed through Field Emission Scanning electron microscope FE-SEM analysis (Hitachi S-4800 & EDX-350, Horiba, Tokyo, Japan). Briefly, the samples were fixed onto a brass holder and coated with osmium tetroxide (OsO_4_) by a VD HPC-ISW osmium coater (Tokyo, Japan) for FE-SEM observation. XRD patterns of BC and BCC composite film were recorded at 40 Kv by an X-Ray diffractometer (X’Pert-APD PHILIPS, Almelo, the Netherlands) using Cu Kα radiation.

### 4.4. Mechanical Testing

To perform the tensile testing, straight-sided samples having dimensions of 200 mm × 25 mm × 1 mm were cut from the air-dried composite sheets of BC and BCC. To prevent compression damages in the jaws of tensile testing machine, aluminum rectangular plates (50 mm × 25 mm) were glued at both ends of the samples. Several quasi-static tensile tests were performed on the BC and BCC samples to compare their mechanical properties. Tensile tests were performed using Instron 8801 servo-hydraulic machine (Norwood, MA, USA) having load cell of 100 kN capacity. Bluehill3 software was used to control the tensile testing machine’s parameters such as loading rate. All the tests were performed at a loading rate of 1 mm/min at ambient conditions and tensile load was applied on samples until their complete failure. To check the accuracy and repetition of the experimental results, for each case three tests were conducted, and test data is the average of these three test readings.

### 4.5. Water Holding and Release Experiments

The water retention time (WRT) and water holding capacity (WHC) experiments were performed for the BCC composites and BC following the reported method [32]. BC and BCC sheets were cut into rectangular pieces (15 cm^2^) and freeze-dried. The freeze-dried samples were weighed and placed in water under ambient temperature for several hrs. After complete wetting (receiving stabilized wet weight), the water retention capabilities of all samples were observed via measuring their weights at varying time intervals until reaching the complete dry state. The WHC was determined by the following formula [32]:

$$\mathrm{WHC}=\frac{\mathrm{Water}\;\mathrm{content}\;\mathrm{removed}\;\mathrm{during}\;\mathrm{drying}\;\left(g\right)}{\mathrm{Dry}\;\mathrm{weight}\;\mathrm{of}\;\mathrm{the}\;\mathrm{sample}\;\left(g\right)}$$

### 4.6. Heavy Metal Absorption

In order to perform the metal absorption study, different metal salt solutions including Zn(NO_3_)_2_, (Cu(NO_3_)_2_, Fe (NO_3_)_3_ and Co(NO_3_)_2_ were prepared in a deionized water, each with a concentration of 10 mg/L (10 ppm). Freeze-dried BC and BCC composites sheets were cut into small pieces of equal size and added into beakers having 200 mL of metal salt solution. Solutions were stirred at 200 rpm for 24 h at 25 °C in shaker. Thereafter, an atomic absorption spectrophotometer (AAS, PinAAcle 500, MedTech Park, Singapore) was used to measure the residual metal ion concentration by analyzing the 10 mL solution from every beaker. For desorption studies, the sheets were removed from the metal salt solutions and gently washed. Then each sample was placed overnight in a flask having 200 mL of distilled water under gentle shaking condition at room temperature. Finally, the above-mentioned protocol was followed for analyzing the desorbed metal ion concentrations.

### 4.7. Invitro-Biocompatibility Determination

Dulbecco’s Modified Eagle’s Medium (DMEM) was used for the culturing of HaCaT cells in 5% CO_2_ at 37 °C. In order to determine cell viability on scaffolds (BC and BCC) were trimmed to round pieces of approximately 20 mm diameter and sterilized under UV. The samples were then seeded with the HaCaT cells at the density of 1.6 × 10^4^ cells/scaffold (BC and BCC) in 12 well plates each having 1 mL of DMEM and incubated for 24 h. The cells seeded on bacterial cellulose were used as a control. The proliferation and cell adhesion were measured using phase contrast microscopic analysis after 48 h of incubation.

### 4.8. Viability Assay of HaCaT

The 3-(4,5-dimethylthiazol-2-yl)-2,5diphenyltetrazolium bromide (MTT) assay was performed to check cell viability on fabricated scaffolds, each scaffold (BC and BCC) of 20 mm diameter was sterilized and then seeded with HaCaT at the density of 4 × 10^4^ cells per scaffolds in 12 well micro titer plate for 1 and 2 days. After the incubation period, samples were rinsed with PBS to remove extra media and unattached cells. 0.5 mg/mL of MTT was added to each well and incubated for an extra 4 h. Extra MTT solution was discarded and dimethyl sulphoxide (DMSO) (200 microliter/scaffold) was added to dissolve the formazan crystals. The optical density values of formazan solution were measured by a microplate reader (Molecular Devices, San Jose, CA, USA) at 540 nm. The cells culture on BC served as control.

## Data Availability

The data presented in this study are available from the corresponding author on request.
